# Impact of donor age and relationship on outcomes of peripheral blood haploidentical hematopoietic cell transplantation

**DOI:** 10.1038/s41409-023-01984-8

**Published:** 2023-04-28

**Authors:** Aaron Pruitt, Feng Gao, Elisa De Togni, Hunter Cochran, Sonia Godbole, Michael Slade, Ramzi Abboud

**Affiliations:** 1 Department of Medicine, Washington University School of Medicine, St. Louis, MO USA; 2 Division of Public Health Sciences, Washington University School of Medicine, St. Louis, MO USA; 3 Division of Oncology, Washington University School of Medicine, St. Louis, MO USA

**Keywords:** Haematological cancer, Stem-cell therapies

## Abstract

Here we describe a retrospective analysis of outcomes in 299 patients who underwent peripheral blood haplo-HCT with PTCy from July 2009 through May 2021 and their association with donor characteristics. Patients had mostly acute leukemias and high or very high DRI. Multivariate analyses were conducted examining OS, NRM, relapse, cytokine release syndrome, acute and chronic GVHD. Donor characteristics included age, sex, relationship, ABO status, CMV status, and HLA match grade. Our analysis revealed increasing donor age was associated with higher NRM (compared to age <30; age 30–44 HR, 1.65; *P* = 0.110, age >44 HR, 1.80; *P* = 0.056) but lower relapse risk (compared to age <30; age 30–44 HR, 0.61; *P* = 0.034, age > 44 HR, 0.71; *P* = 0.132). There were no differences in CRS, aGVHD or cGVHD. We found no difference in outcomes based on the donor-recipient relationship. No differences were found based on HLA match grade or DRB1 match status. Increasing donor age was associated with lower relapse risk but higher NRM, resulting in no difference in OS based on donor age. Other donor factors including relationship (parent/sibling/child/ maternal), CMV status, donor sex, HLA match grade, and DRB1 status were not associated with outcomes.

## Introduction

Allogeneic hematopoetic cell transplant (HCT) is an important therapy for a variety of hematologic malignancies and non-malignant hematologic disorders, and for many patients it represents the only curative intent treatment. An increasingly utilized source for hematopoetic stem cells is HLA haploidentical-related donors [1, 2]. Improvements in GVHD prophylaxis, graft modification, and supportive care have made it a viable alternative to HLA matched sibling or matched unrelated donors [2–7], with shorter time to transplant compared with unrelated donors [8]. Since there are often multiple haploidentical donors available for a given patient, determining which donor characteristics affect transplant outcomes is of particular interest in haploidentical hematopoetic cell transplantation (haplo-HCT).

In the matched-unrelated donor setting, younger donor age is associated with improved transplant-related and non-relapse mortality (NRM) [9–11]. In the haplo-HCT setting, the EBMT has released guidelines to inform donor selection, and as in matched unrelated donors, age has emerged as the selectable donor characteristic with the most evidence in haplo-HCT [12]. Several recent studies have demonstrated improved NRM with younger donors [13–17]. In a large cohort of patients receiving bone marrow-derived grafts and posttransplant cyclophosphamide (PTCy) DeZern et al. even described a benefit in overall survival attributable to improved NRM [14]. A study from the EBMT showed a similar trend toward overall survival benefit when patients over 40 received grafts from younger donors [16]. Interestingly, a multicenter study observed the same phenomenon of better NRM with younger donors, but a simultaneous increase in relapse nullifying any survival benefit [15]. The improved NRM in most of these studies was seen in conjunction with a decrease in acute and/or chronic graft versus host disease (GVHD) with younger donors [13, 14, 18, 19].

There has been more variability in reports regarding the effect of donor relationship/kinship on transplant outcomes. One study suggested improved OS and relapse-free survival (RFS) with children relative to parental donors, though not controlled for donor age [19]. Wang et al. observed better outcomes with paternal rather than maternal donors among parents [17]. Additionally, they and others have noted a correlation between non-inherited maternal antigens (NIMA) and decreased incidence of acute GVHD [17, 20]. However, the largest analyses to date have not demonstrated an effect of donor relationship independent of other donor characteristics [13, 14]. Further, recent studies comparing first-degree and non-first-degree relatives as donors revealed no differences in outcomes between the groups [18, 21].

Other potential variables of interest in selecting a haploidentical donor include degree of HLA disparity, specific HLA allele mismatches, blood group ABO compatibility, and donor/recipient cytomegalovirus (CMV) status. The degree of total HLA disparity has not been shown thus far to be of importance in haplo-HCT [22–24]. However, Solomon et al. did observe that mismatch at the HLA-DRB1 locus as well as killer cell immunoglobulin-like receptor (KIR) - ligand mismatch was associated with improved survival [19]. Their group also described inferior survival with CMV seronegative donors for seropositive recipients. A more recent, larger analysis also noted decreased relapse and improved RFS with HLA-DRB1 mismatching [22].

The graft source in these previously described populations has been predominantly, or in some cases entirely, bone marrow-derived grafts. A uniform population of all peripheral blood grafts has yet to be described. There are important differences in the cellular composition of peripheral blood stem cells (PBSCs) relative to bone marrow, including much higher T cell content, that may alter the biology of HCT. Prior studies have identified higher rates of GVHD with PBSCs [13, 15, 25]. Additionally, their use is extremely common in practice and often preferred by clinicians and patients [1, 25]. We, therefore, evaluated outcomes of peripheral blood haplo-HCT with PTCy at our institution in order to determine the impact of selectable donor characteristics.

## Methods

### Patients, donors, and transplantation procedures

Institutional review board approval was granted for this retrospective study of 324 consecutive patients who underwent haploidentical transplant from July 2009 to June 2021. Donors could be either first-degree or second-degree relatives. Patient-donor pairs who were not both HLA typed at the HLA-A, HLA-B, HLA-DRB1, HLA-C, and HLA-DQB1 loci at a high-resolution level were excluded. In total, 299 patients meet inclusion criteria for the final analysis. Haplo-HCT was performed after either myeloablative or nonmyeloablative conditioning. T-cell-replete peripheral blood grafts were administered on Day 0. GVHD prophylaxis consisted of PTCy on days +3 and +4, mycophenolate mofetil on day +5 to +35, and Tacrolimus on day +5 to +180. A subset of patients received itacitinib on day +3 to day +100 on a clinical trial (NCT03755414).

### Study endpoints and outcome definitions

The primary study outcomes were overall survival (OS), relapse, and non-relapse mortality (NRM). OS was defined as the time from haplo-HCT to death by any cause. RFS was defined as the time from haplo-HCT to death by any cause or relapse, whichever occurred first. NRM was defined as death in the absence of any relapse or progression. Relapse and NRM were considered competing risks. Acute GVHD was graded using the Glucksberg grading system and classified as any (grades I–IV) or severe (grades III–IV) [26]. Chronic GVHD was classified using the National Institutes of Health consensus criteria [27]. Cytokine Release Syndrome (CRS) was graded based on the criteria described by Abboud et al. [28]. ABO mismatch was defined as major, minor, or bidirectional.

### Statistical methods

Patient and donor characteristics were summarized using counts and frequencies for categorical variables or means and standard deviations for continuous variables. The distributions of OS across subgroups of interest were described using Kaplan-Meier product limit methods and compared by log-rank tests. Cumulative incidences of relapse and non-relapse mortality (NRM) were estimated using Gray’s sub-distribution method to account for competing risks. Death without relapse was considered a competing risk for relapse, while relapse was considered a competing risk for NRM. Given that relapse and NRM were in opposing directions for some key variables, the composite endpoint of relapse-free survival was unlikely to be informative and thus not included. Multivariate analyses were performed to assess the association between patient/donor characteristics and outcomes using Cox proportional hazards models for OS and using Gray’s sub-distribution regression for relapse/NRM, while using the backward selection procedure to identify independent prognostic factors. The assumption of proportional hazards was assessed graphically based on residuals out of the corresponding regression models. These potential factors included patient age, disease risk index by Armand criteria, HCT comorbidity index, CMV status, disease status at transplant, and myeloablative or nonmyeloablative conditioning. We included donor age as a continuous variable in the Cox regression model, but a linear relationship was not observed. Therefore, we classified donor age using cutoffs of less than 30, 30–44, and 45 or greater per protocol. We compared parents vs. siblings for patients 40 years of age or younger and children vs. siblings for patients over 40 years of age. All tests were two-sided and significance was set at a *p*-value of 0.05. All the analyses were performed using SAS 9.4 (SAS Institutes, Cary, NC).

## Results

### Patient, donor, and transplant characteristics

In total, 299 patients underwent peripheral blood haplo-HCT and high-resolution HLA typing at the above mentioned loci from July 2009 to June 2021. Mean patient follow up was 20.7 months (0.2–119). Baseline demographics of recipients and donors, as well as other transplant characteristics are shown in Table 1. Median recipient age was 56 (19–74). Most were male (57%) and had AML (66%), while 13% had ALL, 13% MDS/MPN, and 8% other disease classes. Based on the disease risk index (DRI), nearly half of patients (49%) were high or very high, 40% were intermediate risk, and 11% were low risk [15]. Most had an HCT comorbidity index of 3 or greater (74%). The majority of patients were in remission at the time of transplant (63%) and received myeloablative conditioning (51%). Median donor age was 38 (15–71) and 64% were male. The most common donor kinship was child (46%), while 37% were siblings, 12% parents, and 6% were of non-first-degree relation. Among donor-recipient pairs, 34% were both positive for CMV, 26% were both negative, 27% were donor negative-recipient positive, 13% were donor positive-recipient negative. Most patients were a 5 out of 10 HLA match (62%), with 12% matching at the DRB1 locus.

**Table 1:** Baseline patient and donor characterisitcs.

Patient and donor characteristics	
Variable	*N* = 299 (%)
**Patient age**	
Age <46	106 (35.5)
Age 46–65	138 (46.2)
Age >65	55 (18.4)
Median (Standard deviation)	56 (15.7)
Diagnosis^*^	
AML	196 (65.6)
ALL	40 (13.4)
MDS/MPN	38 (12.7)
Other	25 (8.4)
**Disease Risk Index**	
Low	34 (11.4)
Intermediate	120 (40.1)
High/Very high	145 (48.5)
HCT Comorbidity Index^**^	
0–2	79 (26.4)
3 or greater	220 (73.6)
**Disease status at transplant**	
Active	110 (36.8)
Remission	189 (63.2)
**Conditioning**	
Myeloablative	152 (50.8)
Non-myeloablative	147 (49.2)
Karnofsky performance status^***^	
<80	48 (16.1)
80 or greater	250 (83.9)
**Donor age**	
<30	76 (25.4)
30–44	105 (35.1)
>44	118 (39.5)
Median (Standard deviation)	38 (14.2)
**Donor sex**	
Female	108 (36.1)
Male	191 (63.9)
**Relationship category**	
Child	137 (45.8)
Parent	35 (11.7)
Sibling	110 (36.8)
Other	17 (5.7)
**ABO mismatch**	
Major	5 (1.7)
Minor	4 (1.3)
**CMV**^¶^ **match status**	
Donor+/recipient+	99 (33.8)
Donor+/recipient-	39 (13.3)
Donor-/recipient+	79 (27.0)
Donor-/recipient-	76 (25.9)
**HLA**^§^ **match grade**	
5/10	184 (61.5)
>5/10	115 (38.5)
**HLA**^§^ **DRB1 match**	
Yes	34 (11.4)

*AML Acute myeloid leukemia, *ALL* acute lymphoblastic leukemia, *MDS* myelodysplastic syndrome, *MPN* myeloproliferative neoplasm. Other denotes a heterogenous group of transplantable hematologic malignancies not classified elsewhere.

**The hematopoetic cell transplant comorbidity score (HCT CI) is a comorbidity index that comprises 17 different categories of organ dysfunction. Higher scores represent more comorbidity.

***Karnofsky performance status (KPS) is a scale ranging from 0 to 100 with higher scores representing greater functional capacity.

¶Cytomegalovirus (CMV).

§Human leukocyte antigen (HLA).

### Overall survival

**Fig. 1:**
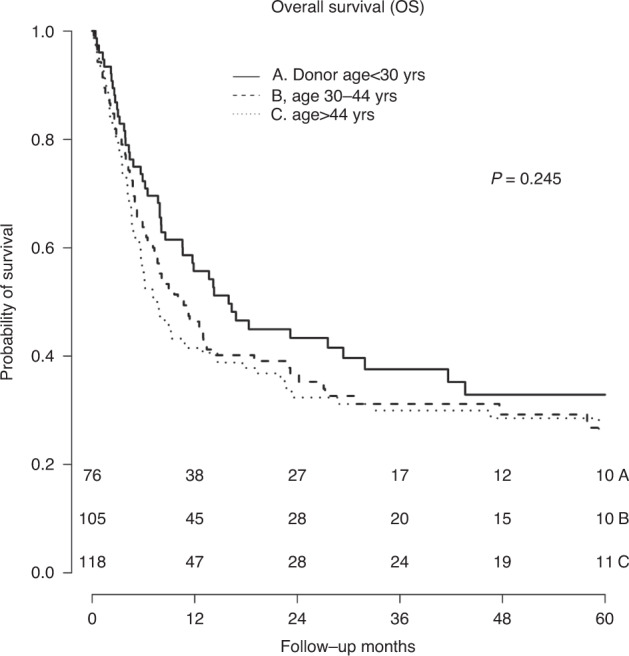
Overall survival by donor age group. Kaplan-Meier curves for overall survival are shown for the three donor age groups. *P*-value is determined by the log rank test.

On univariate analysis (UVA), the following patient and disease related variables were found to be significantly associated with OS (Table 2): patient age (compared to age <46; age 46–65 HR, 1.42; *P* = 0.038, age > 65 HR, 2.42; *P* < 0.001), DRI (compared to low DRI; intermediate HR, 2.26; *P* = 0.011, high/very high HR, 3.34; *P* < 0.001), HCT CI (3 or greater vs. <3; HR, 1.67; *P* = 0.003), KPS > 80 (HR, 0.56; *P* = 0.001), disease status at transplant (remission vs. active; HR, 0.51; *P* < 0.001), and myeloablative conditioning (HR, 0.66; *P* = 0.004). We then evaluated the impact of selectable donor characteristics on survival while adjusting for the above variables. The results of this multivariate analysis are shown in Table 3. Donor age was not associated with OS (compared to age <30; age 30–44 HR, 1.16; *P* = 0.463, age >44 HR, 1.34; *P* = 0.134, Fig. 1). Donor relationship was evaluated as a parent vs. sibling for patients 40 years of age or younger (*n* = 74) and as child vs. sibling for patients >40 years of age (*n* = 199). We further exam maternal donors vs. male donors and non-maternal female donors as well as maternal recipients vs. other female recipients and male recipients. There was no association with OS seen for any relationship category. HLA disparity and HLA DRB1 match status were not evaluated in the multivariate model as they did not reach significance in the univariate analysis.

**Table 2:** Univariate analysis: OS, Relapse, NRM.

Variable	OS		Relapse		NRM	
	HR (95% CI)	*P*	HR (95% CI)	*P*	HR (95% CI)	*P*
**Donor age**						
Age <30	–		–		–	
Age 30–44	1.25 (0.87–1.82)	0.230	0.64 (0.41–0.98)	**0.044**	1.93 (1.07–3.47)	**0.028**
Age >44	1.36 (0.95–1.95)	0.098	0.73 (0.47–1.12)	0.149	1.93 (1.08–3.43)	**0.025**
**Donor sex**						
M	1.27 (0.95–1.71)	0.108	1.09 (0.75–0.60)	0.639	1.17 (0.77–1.77)	0.463
F	–		–		–	
**Donor relationship**						
Child	–		–			
Sibling	0.84 (0.62–1.14)	0.270	0.89 (0.59–1.35)	0.592	0.92 (0.60–40)	0.686
Parent	0.58 (0.35–0.96)	**0.033**	1.06 (0.61–1.85)	0.826	0.60 (0.28–1.28)	0.188
Other	0.83 (0.44–1.54)	0.544	1.44 (0.75–2.77)	0.279	0.64 (0.22–1.86)	0.415
**CMV**^¶^						
D+/R+	–		–		–	
D+/R-	0.93 (0.59–1.48)	0.771	1.50 (0.85–2.67)	0.165	0.64 (0.30–1.37)	0.251
D-/R+	0.96 (0.67–1.36)	0.810	0.94 (0.58–1.53)	0.815	1.13 (0.70–1.81)	0.614
D-/R-	0.72 (0.49–1.05)	0.083	1.10 (0.69–1.75)	0.689	0.80 (0.47–1.36)	0.404
**HLA**^§^ **match**						
5/10	–		–		–	
>5/10	0.86 (0.65–1.15)	0.315	1.26 (0.88–1.80)	0.216	0.65 (0.42–0.99)	**0.049**
**HLA**^§^ **DRB1**						
Match	1.13 (0.74–1.74)	0.567	1.09 (0.62–1.92)	0.765	1.01 (0.53–1.90)	0.990
Mismatch	–					
**Patient age**						
<46	–		–		–	
46–65	1.42 (1.02–.97)	**0.038**	0.68 (0.456–1.01)	0.056	1.93 (1.16–3.21)	**0.011**
>65	2.42 (1.64–3.55)	**<0.001**	1.07 (0.65–1.74)	0.801	2.30 (1.28–4.11)	**0.005**
Diagnosis^*^						
AML	–					
ALL	0.87 (0.56–1.34)	0.516	0.82 (0.47–1.42)	0.473	0.98 (0.52–1.83)	0.947
MDS/MPN	1.16 (0.78–1.74)	0.469	0.62 (0.34–1.15)	0.130	1.90 (1.16–3.10)	**0.005**
Other	0.98 (0.58–1.65)	0.939	1.88 (1.08–3.27)	**0.025**	0.72 (0.32–1.61)	0.419
**DRI**						
Low	–		–		–	
Intermediate	2.26 (1.20–4.26)	**0.011**	1.55 (0.71–3.38)	0.273	1.60 (0.78–3.29)	0.203
High/very High	3.34 (1.80–6.21)	**<0.001**	2.93 (1.38–6.23)	**0.005**	1.52 (0.74–3.13)	0.257
HCI CI^**^						
**<**3	–		–		–	
3 or greater	1.67 (1.19–2.35)	**0.003**	0.77 (0.53–1.14)	0.194	2.98 (1.62–5.47)	**<0.001**
KPS^***^						
<80	–		–		–	
80 or greater	0.56 (0.39–0.80)	**0.001**	1.27 (0.75–2.15)	0.367	0.51 (0.31–0.82)	**0.006**
**Disease status**						
Active	–		–		–	
Remission	0.51 (0.39-–0.68)	**<0.001**	0.42 (0.29–0.60)	**<0.001**	1.001 (0.67–1.51)	0.996
**Conditioning**						
Myeloablative	0.66 (0.50–0.87)	**0.004**	0.62 (0.43–0.89)	**0.009**	0.93 (0.63–1.38)	0.730
Non-myeloablative	–		–		–	

^*^AML Acute myeloid leukemia, *ALL* acute lymphoblastic leukemia, *MDS* myelodysplastic syndrome, *MPN* myeloproliferative neoplasm. Other denotes a heterogenous group of transplantable hematologic malignancies not classified elsewhere.

**The hematopoetic cell transplant comorbidity score (HCT CI) is a comorbidity index that comprises 17 different categories of organ dysfunction. Higher scores represent more comorbidity.

***Karnofsky performance status (KPS) is a scale ranging from 0 to 100 with higher scores representing greater functional capacity.

¶Cytomegalovirus (CMV).

§Human leukocyte antigen (HLA).

Bold values represent variables that reached statistical significance.

**Table 3:** Multivariate analysis.

Variable	OS		Relapse		NRM	
	HR (95% CI)	*P*	HR (95% CI)	*P*	HR (95% CI)	*P*
**Donor age**						
Age <30	–		–		–	
Age 30–44	1.16 (0.78–1.71)	0.463	0.61 (0.39–0.96)	**0.034**	1.65 (0.89–3.05)	0.110
Age >44	1.34 (0.92–1.95)	0.134	0.71 (0.46–1.11)	0.132	1.80 (0.98–3.28)	0.056
**HLA**^§^ **match**						
5/10	–		–		–	
>5/10	–		–		0.70 (0.45–1.09)	0.114
**Patient age**						
Age <46	–		–			
Age 46–65	1.37 (0.97–1.93)	**0.073**	0.68 (0.46–1.02)	0.066	1.67 (0.94–2.94)	0.079
Age >65	2.09 (1.37–3.20)	**<0.001**	1.05 (0.61–1.82)	0.857	1.69 (0.87–3.25)	0.119
**DRI**						
Low	–		–		–	
Intermediate	2.19 (1.16–4,14)	**0.015**	1.49 (0.68–3.26)	0.315	–	
High/Very high	2.23 (1.13–4.40)	**0.021**	1.86 (0.82–4.26)	0.140	–	
HCT CI^**^						
<3	–		–		–	
3 or greater	1.39 (0.98-1.96)	0.064	–		2.64 (1.43-4.90)	**0.002**
**Conditioning**						
Myeloablative	0.82 (0.61-1.10)	0.189	0.53 (0.36-0.77)	**<0.001**	–	
Non-myeloablative	–		–		–	
KPS^***^						
<80	–		–		–	
80 or greater	0.56 (0.39-0.80)	**0.002**	–		0.54 (0.33-0.88)	**0.014**
**Disease status**						
Active	–		–			
Remission	0.55 (0.37-0.80)	**0.002**	0.47 (0.28-0.77)	**0.003**		
Diagnosis^*^						
AML	–		–		–	
ALL	–		–		1.36 (0.70-2.65)	0.368
MDS/MPN	–		–		1.73 (1.05-2.85)	**0.030**
Other	–		–		0.87 (0.42-1.80)	0.702

*AML Acute myeloid leukemia, *ALL* acute lymphoblastic leukemia, *MDS* myelodysplastic syndrome, *MPN* myeloproliferative neoplasm. Other denotes a heterogenous group of transplantable hematologic malignancies not classified elsewhere. **The hematopoetic cell transplant comorbidity score (HCT CI) is a comorbidity index that comprises 17 different categories of organ dysfunction. Higher scores represent more comorbidity. ***Karnofsky performance status (KPS) is a scale ranging from 0 to 100 with higher scores representing greater functional capacity.

¶Cytomegalovirus (CMV).

§Human leukocyte antigen (HLA).

Bold values represent variables that reached statistical significance.

### Relapse

**Fig. 2:**
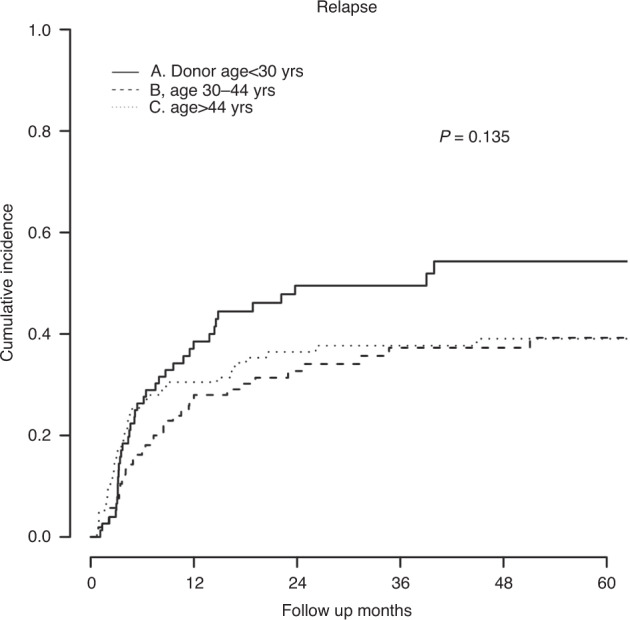
Relapse by donor age group. Cumulative incidence curves for relapse are shown for the three donor age groups. *P*-value is determined by Grayʼs test.

In the UVA, patient, and disease characteristics associated with relapse were DRI (compared to low risk; intermediate HR, 1.55; *P* = 0.273, high/very high HR, 2.93; *P* = 0.005), disease in remission at transplant (HR, 0.42; *P* < 0.001), and myeloablative conditioning (HR, 0.62; *P* = 0.009). Controlling for these variables, the MVA (Table 3) demonstrated increased relapse among the youngest donor age group (compared to age <30; age 30–44 HR, 0.61; *P* = 0.034, age >44 HR, 0.71; *P* = 0.132, Fig. 2). Relapse was not associated with donor relationship, HLA disparity, or HLA-DRB1 matching.

### Non-relapse mortality

**Fig. 3:**
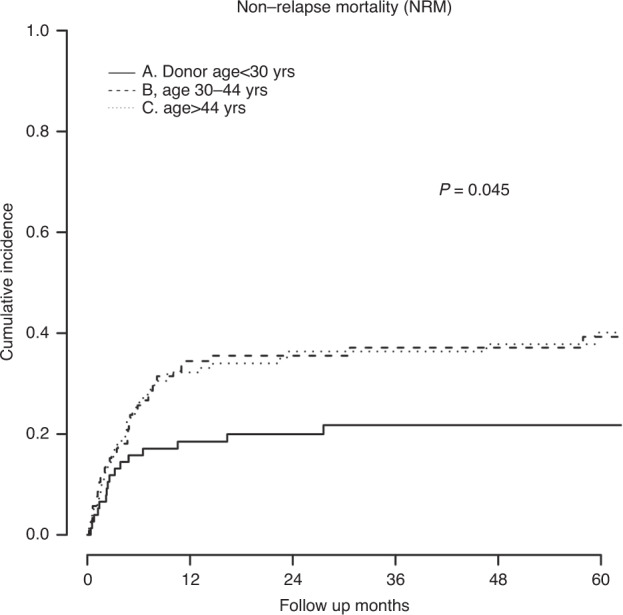
Non-relapse mortality by donor age group. Cumulative incidence curves for non-relapse mortality are shown for the three donor age groups. *P*-value is determined by Grayʼs test.

Significant patient and disease-related variables in the UVA for NRM (Table 2) consisted of patient age (compared to age < 46; age 46-65 HR, 1.93; *P* = 0.011, age >65 HR, 2.29; *P* = 0.005), HCT-CI (3 or greater vs. less than 3; HR, 2.98; *P* < 0.001), and KPS (80 or greater vs. less than 80; HR, 0.51; *P* = 0.006). Subsequent MVA (Table 3) looking at the effects of donor characteristics showed worse NRM with donors older than 30 (compared to age <30; age 30–44 HR, 1.65; *P* = 0.110, age >44 HR, 1.80; *P* = 0.056, Fig. 3). In the univariate analysis an HLA match grade of greater than 5 out of 10 was associated with improved NRM (HR, 0.65; *P* = 0.049), but not in the multivariate model (HR, 0.70; *P* = 0.114). There was no correlation between NRM and donor relationship or DRB1 status.

### CRS and GVHD

No association was found between acute GVHD and any of the examined donor characteristics in the univariate analysis. This was true for both any grade aGVHD and grades III-IV aGVHD. Similarly, no association was seen for any donor characteristics and chronic GVHD or CRS. Thus, a subsequent MVA was not performed.

## Discussion

Haplo HCT represents an important and increasingly utilized therapeutic option for patients undergoing allo-HCT. Patient, disease, and donor related characteristics all contribute to patient outcomes. While patient characteristics and disease biology remain largely unmodifiable, donor characteristics can often be selected for and optimized, especially in haplo HCT where many patients have multiple potential donors. This large, single center, retrospective study of patients receiving peripheral blood stem cell grafts examines the effect of a number of donor characteristics on outcomes, and demonstrates the importance of donor age in choosing the best haplo donor for the clinical situation.

Somewhat surprisingly, our analysis revealed that increasing donor age was associated with higher NRM, but conversely reduced relapse risk. These opposing effects resulted in no net statistically significant difference in OS based on donor age. A trend toward improved overall survival was noted and it is possible that with a larger sample size this effect might reach significance. The deleterious effect of increasing donor age on NRM in haplo HCT has been demonstrated by others, including Canaani et al. and DeZern et al., who found the improvement in NRM with younger donors was such that it conferred a benefit in overall survival [14, 16]. Our study confirms these findings regarding NRM in an all PB population. As others have described [14–16], it is likely GVHD plays a role in this increased NRM with older donors. We did note a trend toward increased chronic GVHD with older donors that did not reach significance in the UVA and thus was not included in the multivariate analysis. As such, it is unlikely this fully explains the observed difference in NRM. Other factors such as delayed graft function, graft failure, and infection are likely contributing and represent areas of potential future investigation.

The reduced risk of relapse with older donors is an unexpected and noteworthy finding of this analysis, though has been described in one other retrospective study by Mariotti et al. [15]. A unique aspect of our cohort was that it utilized peripheral blood grafts as the sole stem cell source, and perhaps this difference played a role in the results with regard to relapse. Other distinctive aspects of our population are high-risk disease biology, high percentage of relapsed or refractory disease at transplant (37%), frequent use of myeloablative conditioning, and high comorbidity. As an example, the group at John’s Hopkins utilized entirely NMA conditioning and 20% had high or very high DRI. In our population, just over half received MA conditioning and 49% had high or very high DRI. It is possible these differences in patient and transplant factors, particularly more high-risk disease and thus a relatively higher risk of relapse, may have potentiated the effect of donor age on relapse in our study. It is worth noting the cohort analyzed by Mariotti et al. approximated ours more closely with the proportion of patients receiving MA conditioning and PB grafts both being nearly 40%, and 37% of patients having high-very high disease risk index.

A clear biological explanation for these observations has yet to be fully elucidated. One possible factor is the increased rate of clonal hematopoiesis of indeterminate potential (CHIP) among older donors. The recent work of Gibson et al. showed donor CHIP, specifically with DNMT3A mutation, was associated with reduced relapse [29]. This could also contribute to the non-linearity of the donor age-relapse relationship, given in their analysis CHIP was present exclusively in donors over 40 years of age. The T cell compartment of grafts may also play a role [30–32]. A study examining donor lymphocyte infusion compositions found older donors had higher CD4/CD8 ratios [30]. There is also some preclinical work suggesting increased age is associated with fewer inducible T regulatory cells relative to naïve T regulatory cells [33, 34]. More work is needed to better understand the mechanisms of the effect of donor age on NRM and relapse.

We did not find that donor relationship had any effect on outcomes after controlling for donor age and patient variables - including age. Additionally, we were interested in the immunologic ramifications of non-inherited maternal antigens. Previously they have been associated with decreased rates of GVHD, and this increased tolerance could plausibly impact relapse and survival as well [17, 20]. Thus, we compared mothers receiving grafts from their children, as well as maternal donors, to all other donor-recipient relationship pairs. No differences were observed in relapse, NRM, GVHD, or other outcomes of interest (Supplemental Figures S1, S2). Our MVA showed a trend toward improved NRM with a higher HLA match grade, though no such trend was seen for overall survival. At this point our data supports that of prior reports suggesting no relationship between degree of HLA disparity and survival in haplo HCT [19, 22, 35]. We did not see an association between HLA DRB1 mismatch and survival or relapse. This was to the contrary of the recent work of McCurdy et al. and could be due to the small number of events observed relative to their registry-based study [22]. Only about 12% of our 299 patient cohort was matched at the DRB1 locus.

We wondered if the observed effects of donor age on NRM and relapse could be explained by correlation with another patient or disease characteristic known to be related to these outcomes, particularly the close association between donor and recipient age. However, in our analysis the correlation between donor and recipient age was modest (correlation coefficient=0.18) and each was able to be reliably assessed in the multivariate models. We carefully controlled for recipient age, donor-recipient relationship, sex, HCT-CI, DRI, disease status, conditioning regimen, CMV, and KPS and found that donor age remained a significant and independent predictor of NRM and Relapse. Additionally, NRM and relapse were evaluated using the Gray’s sub-distribution method to account for their competing risk. Our study has a number of limitations. While it benefits from uniformity in graft source, it is smaller than the multicenter and registry-based studies traditionally used for donor optimization. Furthermore, this is a single center, retrospective analysis. However, this did allow for granular data collection and uniform transplant protocols.

In conclusion, increasing donor age was associated with higher NRM. However, relapse risk was lower with donors over the age of 30. These competing effects resulted in no significant difference in overall survival based on donor age. Our data suggests that in peripheral blood haplo-HCT, younger donors may be preferred in patients with high risk of transplant related complications. A unique finding of our analysis is the reduction in relapse we observed when using older donors. Further exploration of this effect is warranted in larger, multicenter studies, particularly in populations where relapse risk is high.

## Supplementary information

Supplemental Figure 1

Supplemental Figure 2

Supplementary Figure Legends

## Data Availability

The datasets generated during and/or analyzed during the current study are not publicly available, but are available from the corresponding author on reasonable request.
