# Supplementary figures and images for: Impact of donor age and relationship on outcomes of peripheral blood haploidentical hematopoietic cell transplantation
Source: Bone Marrow Transplant. 2023 Apr 28;58(8):855–62. doi: 10.1038/s41409-023-01984-8 (PMC10400423; doi:10.1038/s41409-023-01984-8)

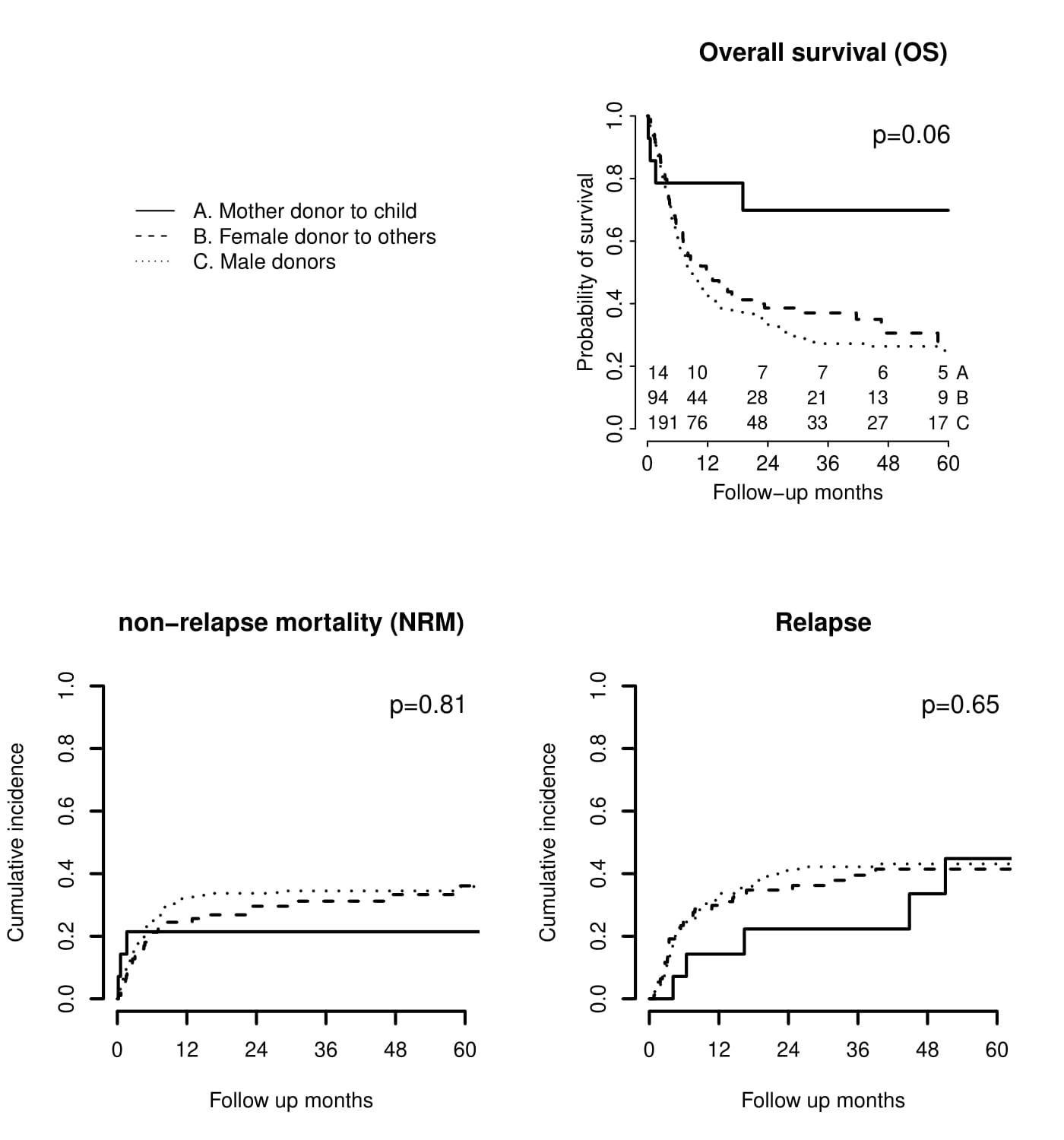

Supplement: Supplementary file 1 — Supplemental Figure 1 [file 41409_2023_1984_MOESM1_ESM.jpg]

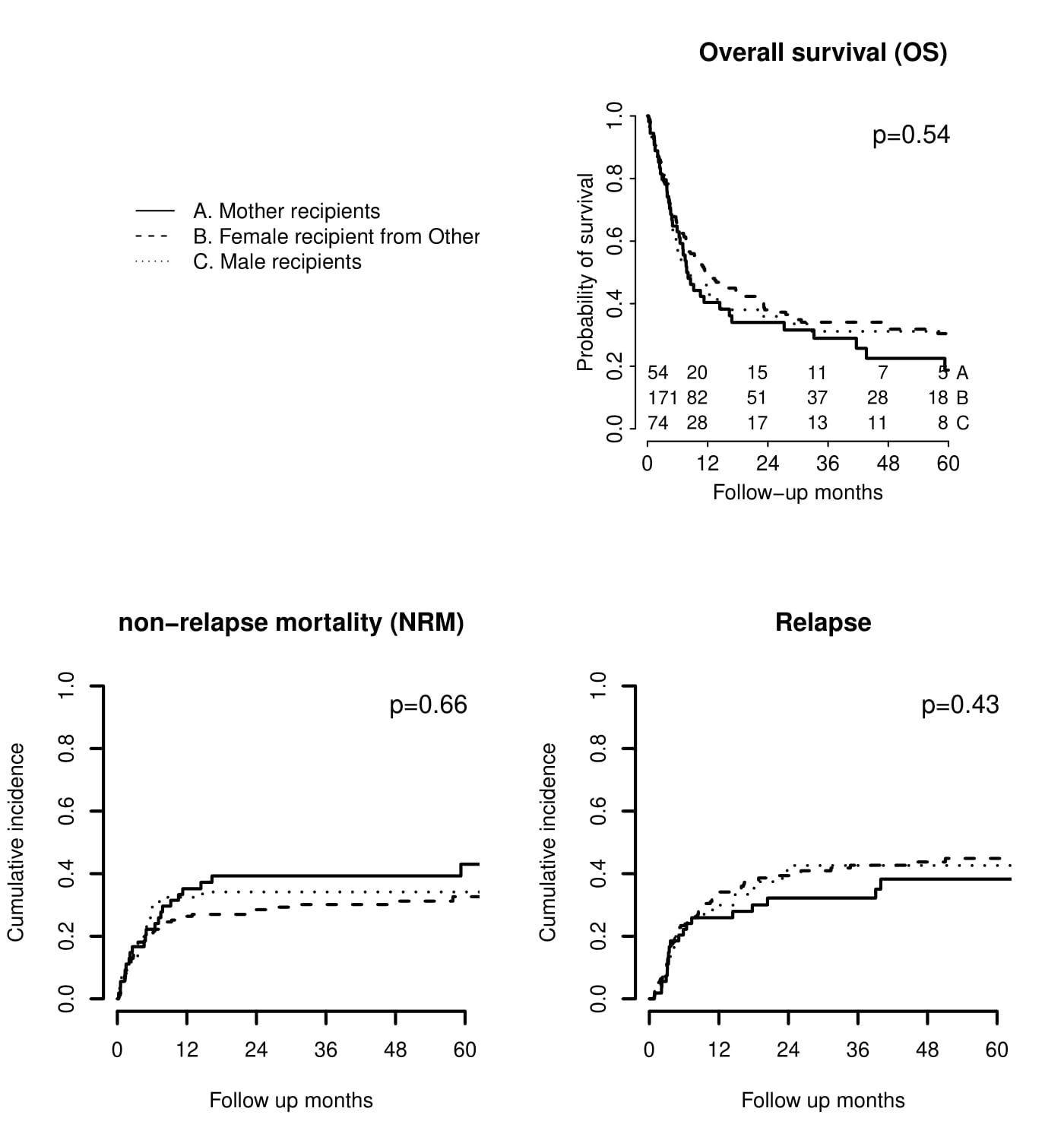

Supplement: Supplementary file 2 — Supplemental Figure 2 [file 41409_2023_1984_MOESM2_ESM.jpg]
