# Supplementary material for: Impact of donor age and relationship on outcomes of peripheral blood haploidentical hematopoietic cell transplantation
Source: Bone Marrow Transplant. 2023 Apr 28;58(8):855–62. doi: 10.1038/s41409-023-01984-8 (PMC10400423; doi:10.1038/s41409-023-01984-8)
Supplement: Supplementary file 3 — Supplementary Figure Legends [file 41409_2023_1984_MOESM3_ESM.docx]

**Supplementary Figure Legends:**

**Figure S1.** Kaplan-Meier curves for overall OS and cumulative incidence curves for relapse and NRM for maternal donors compared to other female donors and male donors. P values based on log-rank test for OS and Gray’s test for relapse and NRM.

**Figure S2.** Kaplan-Meier curves for overall OS and cumulative incidence curves for relapse and NRM for maternal recipients from their children compared to other female recipients and male recipients. P values based on log-rank test for OS and Gray’s test for relapse and NRM.
